# Saccade-induced image motion cannot account for post-saccadic enhancement of visual processing in primate MST

**DOI:** 10.3389/fnsys.2015.00122

**Published:** 2015-09-01

**Authors:** Shaun L. Cloherty, Nathan A. Crowder, Michael J. Mustari, Michael R. Ibbotson

**Affiliations:** ^1^National Vision Research Institute, Australian College of OptometryCarlton, VIC, Australia; ^2^Department of Optometry and Vision Sciences, Australian Research Council Centre of Excellence for Integrative Brain Function, University of MelbourneParkville, VIC, Australia; ^3^Department of Electrical and Electronic Engineering, University of MelbourneParkville, VIC, Australia; ^4^Department of Psychology and Neuroscience, Life Sciences Centre, Dalhousie UniversityHalifax, NS, Canada; ^5^Visual Sciences, Yerkes National Primate Research Center, Emory UniversityAtlanta, GA, USA

**Keywords:** visual cortex, macaque monkey, medial superior temporal area, electrophysiology, ocular following, eye movements

## Abstract

Primates use saccadic eye movements to make gaze changes. In many visual areas, including the dorsal medial superior temporal area (MSTd) of macaques, neural responses to visual stimuli are reduced during saccades but enhanced afterwards. How does this enhancement arise—from an internal mechanism associated with saccade generation or through visual mechanisms activated by the saccade sweeping the image of the visual scene across the retina? Spontaneous activity in MSTd is elevated even after saccades made in darkness, suggesting a central mechanism for post-saccadic enhancement. However, based on the timing of this effect, it may arise from a different mechanism than occurs in normal vision. Like neural responses in MSTd, initial ocular following eye speed is enhanced after saccades, with evidence suggesting both internal and visually mediated mechanisms. Here we recorded from visual neurons in MSTd and measured responses to motion stimuli presented soon after saccades and soon after simulated saccades—saccade-like displacements of the background image during fixation. We found that neural responses in MSTd were enhanced when preceded by real saccades but not when preceded by simulated saccades. Furthermore, we also observed enhancement following real saccades made across a blank screen that generated no motion signal within the recorded neurons' receptive fields. We conclude that in MSTd the mechanism leading to post-saccadic enhancement has internal origins.

## Introduction

Humans and their primate cousins make rapid, pre-planned eye movements approximately three times per second. These eye movements, known as saccades, serve to direct the eyes toward features of interest. Saccades are made frequently when freely viewing natural scenes (Yarbus, 1967) and it has been established that saccade frequency increases when viewing novel stimuli, suggesting a visual processing advantage (Buswell, 1935; Yarbus, 1967; Antes, 1974). The potentially distracting image motion generated by saccades is perceptually suppressed (Diamond et al., 2000; Thiele et al., 2002; Price et al., 2005; Ibbotson et al., 2008; Bremmer et al., 2009; Ibbotson and Cloherty, 2009) and there is considerable evidence for a concomitant attenuation, but not a complete suppression, of spiking activity in the visual pathways during saccades (Thiele et al., 2002; Price et al., 2005; Ibbotson et al., 2008; Bremmer et al., 2009; Ibbotson and Cloherty, 2009; for review: Ibbotson and Krekelberg, 2011). Moreover, behavioral evidence suggests that performance of the visual system is enhanced immediately after saccades. For example, reflexive ocular following responses have higher initial speeds immediately after saccades (Kawano and Miles, 1986; Ibbotson et al., 2007). At the neural level, a series of recent discoveries suggests a tight coupling between saccadic eye movements and the processing of visual information (Ibbotson and Krekelberg, 2011). Single-unit recordings in primates have shown that responses to visual stimulation are enhanced following a saccade compared to those during fixation. This enhancement is evident in recordings from the lateral geniculate nucleus (LGN) of the thalamus (Reppas et al., 2002; Royal et al., 2006), primary visual cortex (V1, Kagan et al., 2008; Rajkai et al., 2008) and parietal cortex (Ibbotson et al., 2007, 2008; Bremmer et al., 2009; Ibbotson and Cloherty, 2009; Cloherty et al., 2010) and persists for several hundred milliseconds after a saccade. Furthermore, studies of synchronous activity between neurons in V1 show increased correlations following saccadic eye movements (Maldonado et al., 2008). Importantly, this increase in correlated activity precedes the post-saccadic increase in spike rate, suggesting that in addition to response amplitude, spike timing may also play a key role in inter-saccadic processing of visual information. In accordance with this, neural responses to visual stimuli at the time of saccades have shorter latencies (Price et al., 2005; Ibbotson et al., 2006). Combined, the available evidence suggests that saccadic eye movements do more than simply point the eyes in the right direction; they also appear to set off a cascade of neural changes important for maximizing performance of the visual system.

There are two possible mechanisms for the observed post-saccadic enhancement of neural activity. An internal mechanism associated with saccade planning and execution, a so-called *corollary discharge*, could sensitize visual areas of the brain. Alternatively, rapid motion of the retinal image during saccades could activate gain control mechanisms that transiently enhance visual sensitivity. Post-saccadic enhancement of ocular following eye movements is influenced by both mechanisms: a strong motion signal during a saccade potentiates the internally generated enhancement (Kawano and Miles, 1986). At the neural level it has been established that post-saccadic enhancement occurs even for saccades in darkness. However, this non-visual enhancement occurs sooner than for saccades in the light, suggesting that a different mechanism may underlay this effect (Cloherty et al., 2010). In the present study we recorded visual responses from neurons in the dorsal medial superior temporal (MSTd) area of macaque cortex. We presented moving test stimuli soon after saccades and soon after simulated saccades—saccade-like displacements of a high contrast background texture during fixation. We found that responses to the test stimuli were enhanced soon after real saccades but not after simulated saccades, suggesting that post-saccadic enhancement in MSTd may be attributed primarily to an internal as opposed to a visual mechanism.

## Materials and methods

### Surgical procedures

Data were collected from two juvenile male rhesus monkeys (*Macaca mulatta*). All surgical and experimental procedures were performed in compliance with National Institutes of Health guidelines and protocols approved by the Institutional Animal Care and Use Committee at Emory University. An MRI compatible head stabilization system (Crist Instruments, Hagerstown, MD USA) and a recording chamber were stereotaxically implanted over the superior temporal sulcus (lateral, 15 mm; posterior, 5 mm) under aseptic conditions using isoflurane anesthesia (1.25–2.0%). A scleral search coil for measuring eye movements was implanted underneath the conjunctiva of both eyes.

Locations of recording sites were confirmed using a Siemens 3-Tesla magnetic resonance imager (MRI) located at the Yerkes National Primate Research Center. Imaging sessions to acquire 3-dimensional T1-weighted images were performed under sedation (ketamine/telezol) and surgical levels of isoflurane (1.0–1.5%). Vital signs including blood pressure, heart rate, body temperature, expired CO_2_, and blood oxygenation were continuously monitored and maintained at physiological levels. Monkeys were held in an MRI compatible stereotaxic frame (Crist Instruments, Hagerstown, MD USA) during imaging sessions. Scan parameters were set to sample 1 mm slices through the entire anterior–posterior extent of the brain including the recording chambers mounted over MSTd. We used Neurolens software to identify regions of interest below our Cilux recording chambers (Crist Instruments, Hagerstown, MD USA).

Electrodes for single unit recordings were placed into MSTd using guide tubes precisely positioned using an adjustable radius-and-angle positioning device attached to the recording chamber. For visualization during MRI sessions the positioning device was fitted with a centering bushing that carried a saline-filled guide tube made of fused silica (Plastics One, Roanoke, VA USA). The small internal diameter (0.15 mm) of the fused silica probe facilitated accurate localization. Our recording tracks vertically penetrated the anterior bank of the superior temporal sulcus and entered area MSTd. All recording sites in both monkeys were in dorsal MST.

### Visual stimuli and task

Visual stimuli and fixation targets were rear projected onto a tangent screen placed 61 cm in front of the animals covering a visual angle of 77° × 77° Stimuli were projected using a Mirage 2000 Digital Light Projector (Christie Digital, Cypress, CA USA) with resolution 1024 × 1024 pixels, frame rate 96 Hz and mean luminance 170 cd/m^2^ (Price et al., 2005). The Mirage 2000 does not suffer from typical refresh and scanning problems associated with CRT or LCD displays (for details see Ibbotson et al., 2008).

Monkeys were comfortably seated with their head stabilized in the horizontal stereotaxic plane and received a fruit juice reward every 0.5–1 s for maintaining fixation on a red target presented on the screen. Before the experiment, we determined the preferred direction and speed of a cell using moving texture patterns, while the animal fixated the target. We also used a small patch of moving dots that could be placed at any location on the screen. The dots in the patch moved in the cell's preferred direction. The patch was moved around the screen to establish the borders of the receptive fields. We present data only from those cells for which we could locate the edge of the receptive field and for which the receptive field remained within the bounds of the screen at all times.

**Figure 1:**
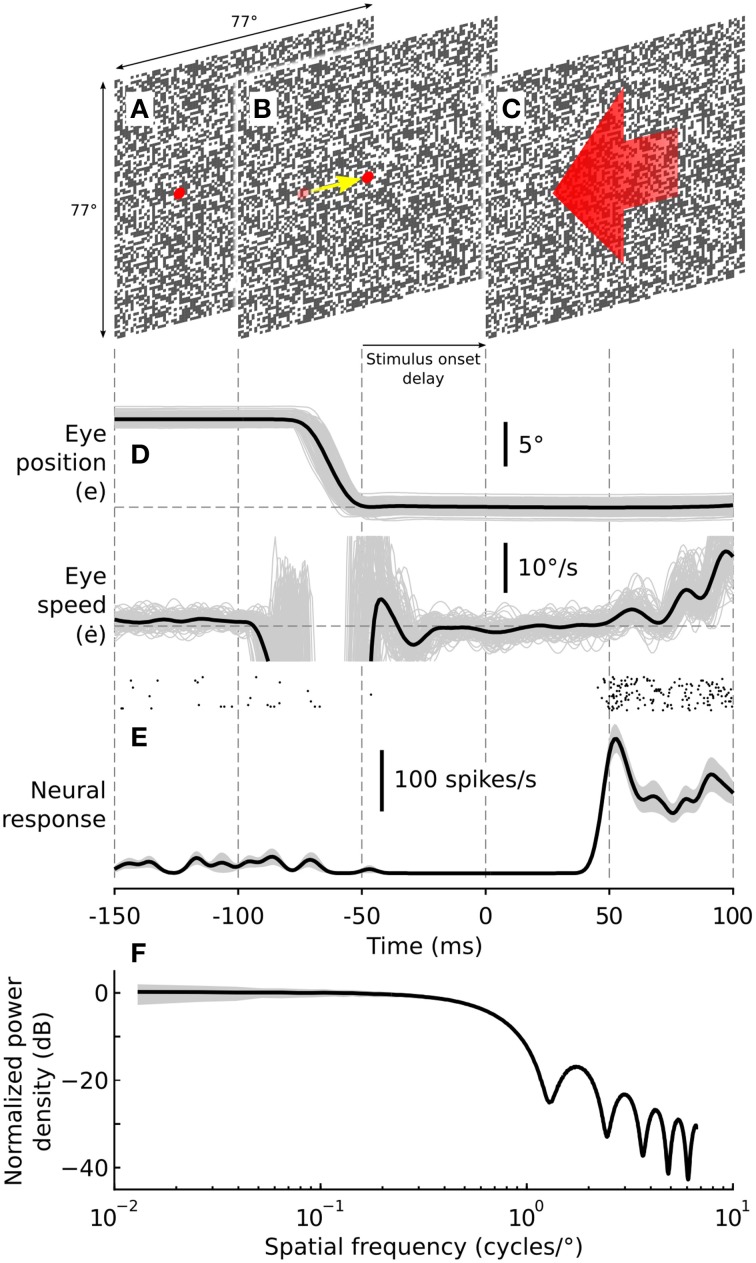
**Experimental methodology**. Visual stimuli consisted of a random checkerboard texture, 77° × 77° with a check size of 0.8° × 0.8°. Monkeys made rewarded saccades from a peripherally presented target **(A)** to a centrally presented target **(B)**. After the centering saccade the monkeys were required to maintain fixation for a nominal period of either 50 (short-delay condition) or 300 ms (long-delay condition) after which the background texture began moving **(C)**, eliciting robust spiking responses and triggering reflexive ocular following. The post-saccadic test stimulus always moved in the preferred direction (opposite to that of the saccade) and at the preferred speed of the recorded cell. Peri-saccadic stimuli were presented at 100% contrast, and for some cells, at 0% contrast to determine the effect of visual stimulation during the saccade on the observed post-saccadic enhancement. Post-saccadic motion stimuli were always presented at 100% contrast. **(D)** Eye movements were recorded using scleral search coils implanted in both eyes. Eye speed of the reflexive ocular following response was calculated off-line, providing a behavioral assay for post-saccadic enhancement. Thin gray lines show eye position and speed for individual trials. The black lines show representative eye position and speed signals averaged across all trials. **(E)** Spiking activity was recorded from visual neurons in the dorsal part of the medial superior temporal area (MSTd). The raster plot shows spike arrival times from a representative neuron for all trials (*n* = 20). The black line shows the mean spike density function while the shaded region shows ±1 SE. **(F)** Normalized orientation averaged power spectrum of the post-saccadic test stimulus. The black line shows the power spectrum averaged over 1000 random texture samples, normalized to the mean power at 0.1 cycles/°. The shaded region shows the standard deviation over the random texture samples. The power spectrum is flat up to approximately 0.625 cycles/°, falling off gradually thereafter with increasing spatial frequency. At 0.1 cycles/° the stimulus has an orientation averaged power spectral density equal to the RMS contrast power of a cosine grating with Michelson contrast 57.8%.

During the experiment, monkeys initially fixated a peripheral target superimposed on a random background texture (Figure 1A). The peripheral target was presented 10° from the center of the screen, on the circumference of an imaginary circle (radius = 10°) centered on the screen. The background texture covered the entire screen and consisted of 0.8° black and white squares (Figures 1A–C). These textures were used because they provide a broad spectrum of spatial frequencies (Figure 1F) such that any spatial frequency specific effects were unlikely to influence our results. Once the monkey fixated the peripheral target, the peripheral target was removed and the central target was presented (Figure 1B). The monkeys subsequently made a 10° centering saccade to this target across the stationary background, thus generating saccade-driven image motion across the retina. Once the indicated eye position arrived at the central target the monkeys were required to maintain fixation for an interval of either 50 or 300 ms for a juice reward. These two conditions are referred to below as the short-delay and long-delay conditions, respectively. After the prescribed delay, the central fixation target was removed and the background texture simultaneously moved in the preferred direction of the recorded cell at the cell's preferred speed for a period of 150 ms (Figure 1C; short-delay condition shown). In the following we refer to this period of motion of the background texture as the *test* stimulus. The post-saccadic test stimulus always moved in the preferred direction (opposite to that of the saccade) and at the preferred speed of the recorded cell.

In addition to the saccade paradigm just described, we presented the same test stimulus after simulated saccades. The temporal sequence of the stimulus in this paradigm was identical to that just described for real saccades but the monkeys were required to fixate a central target while the background texture (100% contrast) was displaced with a saccade-like trajectory (as outlined in Price et al., 2005). Such simulated saccades generated the same visual motion signal within the recorded cell's receptive field as experienced during a real saccade but without the monkey planning or executing a saccade.

For some cells we had the monkeys perform a third paradigm in which they performed real saccades (as in the saccade paradigm above) with the contrast of the stationary background texture present during the saccade set to 0% (an isoluminant gray screen matched to the mean luminance of the test stimuli). In this condition the saccade was made over a uniform gray screen, eliminating the saccade generated visual motion signal within the recorded cell's receptive field. In all three paradigms, the post-saccadic test stimuli were presented at 100% contrast.

During the test stimulus there was no fixation target present. Nevertheless, the monkeys typically maintained their eye position for approximately 50 ms before the onset of reflexive tracking eye movements. This period is referred to as the open-loop phase and is caused by the inherent latency in the ocular following system. That is, although the test stimulus is moving, it takes around 50 ms for the signals to be processed and for the eye muscles to start moving the eye and thus influence the speed of the retinal image motion. In cases where eye movements occurred during either the stimulus delay or the open-loop phase the trial was omitted from the analysis presented below. Example eye position (e) and eye speed (ė) signals from one monkey are shown in Figure 1D. The evoked neural response recorded from an MSTd neuron is shown as a raster plot and as a spike density function (SDF) below the eye movement signals (Figure 1E). It is evident in this example that the cell's spontaneous activity was reduced after the saccade and that the test stimulus presented 50 ms after saccade-end generated a robust spiking response.

### Data collection

Eye position in two dimensions was measured using a magnetic coil system (CNC Electronics) and sampled at 1 kHz. For timing of the post-saccadic motion stimulus onset delay, saccade end was defined as that point when the eye position came within 1° of the saccade target. Noise in the eye position signals gave rise to some temporal jitter in the timing of the post-saccadic stimulus onset delay. In the short-delay condition, nominally 50 ms, delays ranged from 50 to 109 ms with a mean delay of 82 ms (±10.9 ms, SD). In the long-delay condition, nominally 300 ms, delays ranged from 300 to 360 ms with a mean delay of 332 ms (±11.2 ms, SD).

Spiking responses were recorded from neurons in the dorsal medial superior temporal area (MSTd) of the parietal cortex, a region known to be involved in processing global visual motion (Duffy and Wurtz, 1997), to play a direct role in visual motion perception (Newsome and Paré, 1988) and to be correlated with reflexive ocular following responses (Kawano, 1999). Single-unit activity was measured using iron tipped, epoxy-coated tungsten electrodes (Frederick-Haer Corporation, Bowdoin, ME USA) with impedances ranging from 1 to 4 MOhm. The signal from the electrode was amplified, sampled at 25 kHz and stored for offline analysis. Action potentials were detected online with an analog window discriminator (Alpha-Omega, Alpharetta, GA USA). The output from the window discriminator together with saccade target and stimulus timing signals were logged as event markers in register with the eye position and unit activity signals. All signals were digitized with 16-bit precision using a Power 1401 acquisition system (CED, Cambridge, UK).

### Data analysis

Eye velocity was calculated off-line using a low-pass digital differentiating filter (*N* = 32, low-pass cut-off 80 Hz). Onset of the post-saccadic test stimulus was determined by way of a frame synchronous event marker generated by the stimulus computer. Spike arrival times were determined off-line through action potential template matching (Spike2; CED, Cambridge, UK). Neural responses were represented as spike density functions (SDFs) with 1 kHz resolution generated by convolution of a Gaussian kernel of unit area and σ = 3 ms with a train of Dirac delta functions, one delta function corresponding to the arrival time of each spike. Mean SDFs were then calculated by averaging responses to individual stimulus presentations. For each cell, the spontaneous rate was estimated based on the mean spike rate averaged across periods between trials during which the monkeys maintained fixation on a target presented on an isoluminant gray screen. Spontaneous rate was estimated for each cell by averaging across multiple periods of 200 ms duration (in all cases, *n* ≥ 36).

Neural response latencies were calculated relative to stimulus onset using a Poisson analysis of the spike rate. Spike rates were compared with a Poisson distribution fitted to the intrinsic spontaneous rate to identify periods of significant modulation. Response latency was defined as the beginning of the first period of significant modulation during which the spike rate exceeded for at least 25 ms the 99% cut-off of the Poisson distribution fitted to the spontaneous rate.

For each recorded cell, we then calculated the mean spike rate in the first 50 ms after response onset. The set of neural responses evoked by the test stimulus in multiple trials of the short- and long-delay conditions were denoted *r*_*S*_ and *r*_*L*_, respectively. To quantify post-saccadic enhancement we calculated an enhancement index (EI) for each cell according to:

(1) $$EI=\;\frac{\bar{r_{S}}-\bar{r_{L}}}{\bar{r_{S}}+\bar{r_{L}}}$$

where r_S_ and r_L_ denote the sample means of the set of observed responses evoked in the short- and long-delay conditions, respectively.

Ocular following responses evoked by the test stimulus, presented after either real or simulated saccades, were analyzed separately for each combination of test speed and direction. For each trial corresponding to a given speed and direction of the test stimulus we calculated the initial peak eye speed of the reflexive following response. Onset of the following response was defined as the earliest time after stimulus onset at which the eye acceleration in the direction of the stimulus exceeded 100°/s^2^. Initial peak eye speed was then defined as the eye speed corresponding to the first subsequent negative going zero crossing of the eye acceleration signal. To quantify enhancement of ocular following eye speed we again calculated an enhancement index (EI) as per Equation (1), however, in this case r_S_ and r_L_ denote the sample means of the set of observed ocular following eye speeds for the short- and long-delay conditions, respectively.

### Statistics

#### Neural responses

To evaluate the significance of any observed enhancement in a given cell, we performed a bootstrap hypothesis test (Efron and Tibshirani, 1993). Having calculated an enhancement index (*EI*) using the set of observed responses (i.e., *r*_*S*_ and *r*_*L*_) we then calculated the probability of observing an *EI* at least that large if the mean μ_*S*_ of the distribution from which the *r*_*S*_ were drawn was identical to the mean μ_*L*_ of the distribution from which the *r*_*L*_ were drawn. Specifically, we converted our data set into one which obeyed the null hypothesis (H0: μ_*S*_ = μ_*L*_) by calculating ${\tilde{r}}_{S}=r_{S}-\bar{r_{S}}+\bar{r},\text{ and }\bar{r_{L}}=r_{L}-\bar{r_{L}}+\bar{r}$ where r denotes the mean of the combined sample. We then drew random samples ${\tilde{r}}_{S}{}^{*}\text{and }{\tilde{r}}_{L}{}^{*}$, with replacement, from ${\tilde{r}}_{S}\text{ and }{\tilde{r}}_{L}$ and recalculated the enhancement index (EI^*^). This sampling procedure was repeated 10,000 times providing a distribution of EI^*^s from which we estimated the *achieved significance level* (ASL) of the test as the percentage of samples for which EI^*^ exceeded the observed *EI*. The smaller the ASL, the greater the evidence against the null hypothesis. We rejected the null hypothesis only if the ASL < 0.05.

To test for enhancement of neural responses following real and simulated saccades we again performed a bootstrap hypothesis test on the neural enhancement indicies (*EI*s). Specifically, we calculated the mean *EI* over our cell population for both real and simulated saccades. Using a resampling procedure analogous to that described above, we then calculated the probability of observing *EI* at least that large if the mean of the distribution from which the *EI*s were drawn was zero. Again, we rejected the null hypothesis only if the ASL < 0.05.

To test for differences in responses for the long-delay condition across our three paradigms we performed a non-parametric One-Way analysis of variance (ANOVA; Kruskal-Wallis test) on the neural responses across all cells in our population. We then used post-hoc rank sum tests (Dunn's procedure), with Sidak's correction for multiple comparisons, to identify significant pair-wise differences.

#### Ocular following

As noted above, ocular following responses evoked by the test stimulus were analyzed separately for each combination of speed and direction. To evaluate the significance of any observed enhancement we performed a bootstrap hypothesis test analogous to that described above for the neural responses.

## Results

We had monkeys make rewarded saccades between two alternately presented fixation targets, one peripheral and one central to the stimulus screen. We measured spiking responses in area MSTd and reflexive ocular following eye movements evoked by post-saccadic motion of a high contrast background texture. To investigate the possible role of planning and executing a saccade in modulating post-saccadic enhancement we also had the monkeys fixate a central target while we displaced the background texture with a saccade-like trajectory (a simulated saccade). In effect preserving the nature and timing of the visual input to the recorded cells associated with the real saccades but without the monkeys planning or executing an eye movement.

### Enhancement of neural responses

**Figure 2:**
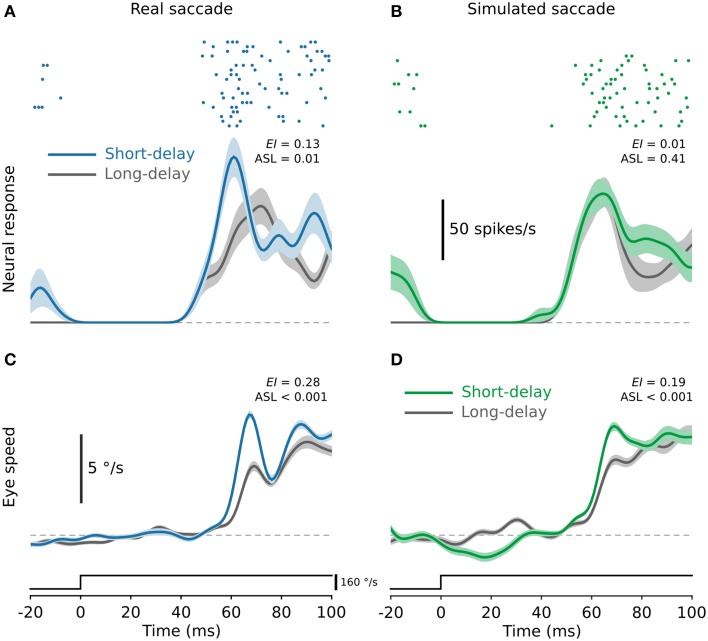
**Post-saccadic enhancement of neural responses and ocular following eye speed. (A)** Representative neural responses from a single MSTd neuron for test stimuli delivered after real saccades. Responses for the short-delay condition (blue) are significantly enhanced compared to those for the long-delay condition (gray). **(B)** Responses from the same neuron for the same test stimulus delivered after simulated saccades. There is no evidence of enhancement of neural responses after simulated saccades. In both **(A,B)** the raster plots show responses for the short-delay condition. **(C,D)** Representative ocular following eye speeds for the same test speed for which the recordings in **(A,B)** were obtained. Ocular following eye speed is significantly enhanced in the short-delay condition compared to the long-delay condition following both real **(C)** and simulated **(D)** saccades. In all panels, solid lines show the average across all trials of a given condition and the shaded regions indicate ±1 SE, estimated by bootstrapping.

**Figure 3:**
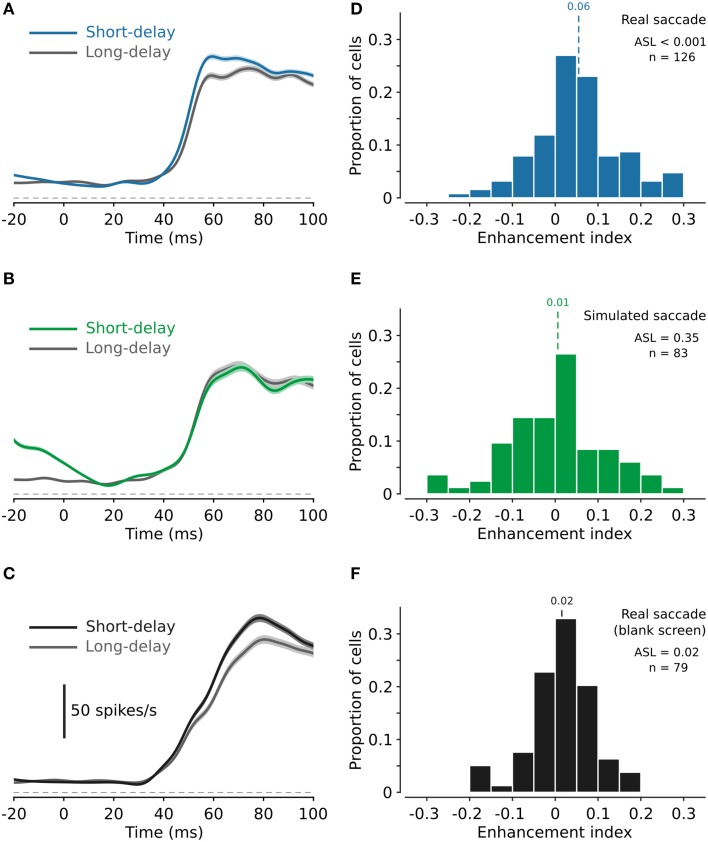
**Enhancement of neural responses after real and simulated saccades, and the effect of peri-saccadic stimulus contrast. (A,B)** Neural responses for test stimuli presented after real and simulated saccades, respectively, averaged across all cells. Responses are enhanced after real saccades **(A)** but not after simulated saccades **(B)**. **(C)** Neural responses for test stimuli presented after real saccades made across a blank screen (i.e., contrast of the background texture during the peri-saccadic period was 0%). As for responses following saccades across the high contrast background texture, neural responses for this paradigm are on average greater in the short-delay condition compared to the long-delay condition. **(D,E)** Histograms of the neural enhancement index, for all cells, for test stimuli presented after real and simulated saccades, respectively. On average, responses are significantly enhanced after real saccades [**(D)**, mean enhancement index 0.06, ASL < 0.001; *n* = 126] but not after simulated saccades [**(E)**, mean enhancement index 0.01, ASL = 0.35; *n* = 83]. **(F)** Histogram of the neural enhancement index for test stimuli presented after real saccades made across a blank screen. For this paradigm the enhancement index reveals a small but significant enhancement of the neural responses (mean enhancement index 0.02, ASL = 0.02, *n* = 79).

Figure 2A shows neural responses from a single cell for both the long- (300 ms, gray) and short-delay (50 ms, blue) conditions. For this cell neural responses to test stimuli delivered soon after real saccades (i.e., the short-delay condition) were significantly enhanced (Figure 2A; ASL = 0.01). In contrast, neural responses to the test stimuli were unchanged following simulated saccades (Figure 2B; ASL = 0.41). Moreover, there was no significant difference in the responses for the long-delay condition following real as opposed to simulated saccades (ASL = 0.4). This is representative of the cells in the broader population. In total we tested 145 MSTd neurons from two monkeys, although not all cells were tested in all conditions (hence the differing cell counts in the population data presented below). We found no significant difference between animals for any of the conditions tested (rank sum tests, *P* > 0.05). In all cases, cells from both animals were therefore combined and analyzed as a single population. Figures 3A,B show neural responses averaged across all cells, for both the long- and short-delay conditions, using the same conventions as in Figure 2. Figure 3C shows responses averaged across all cells for test stimuli delivered after real saccades made over a blank screen.

For each recorded cell, we calculated the mean spike rate in the first 50 ms after response onset (see Methods for criteria used to determine response onset) and thereby calculated for each cell an enhancement index (*EI*; see Methods). Figure 3D shows the distribution of *EI*s calculated from the spiking responses of 126 MSTd neurons for test stimuli delivered after real saccades. Across the cell population, neural responses to the test stimulus were significantly enhanced after saccades (*EI* = 0.06; ASL < 0.001). Figure 3E shows an analogous distribution of *EI*s (*n* = 83) for test stimuli delivered after saccade-like image displacements (i.e., simulated saccades). On average, neural responses were unaffected by simulated saccades (*EI* = 0.01; ASL = 0.35). Figure 3F shows the distribution of *EI*s (*n* = 79) for test stimuli delivered after real saccades made over a blank screen (i.e., a background texture of 0% contrast). On average, neural responses were again enhanced (*EI* = 0.02; ASL = 0.02), although notably the apparent level of enhancement was less (*EI* = 0.02 compared with *EI* = 0.06; ASL = 0.001; see Discussion).

To verify that the increase in *EI*s following saccades is due to enhancement of responses in the short-delay condition and not due to differences in the responses for the long-delay condition, we compared neural responses for the long-delay condition across our three paradigms. A non-parametric One-Way ANOVA revealed a significant difference in response amplitude for the long-delay condition across our three paradigms (Kruskal-Wallis test, *P* = 0.003). Specifically, responses for the long-delay condition were greater following real saccades over the blank screen compared to those following both real saccades with the high contrast background texture (rank sum test, *P* = 0.002) and simulated saccades (rank sum test, *P* = 0.004). This is evident in the population averages shown in Figures 3A–C. However, there was no significant difference in responses for the long-delay condition following real and simulated saccades with the high contrast background texture (rank sum test, *P* = 0.6). Confirming that the increased *EI*s observed for real saccades (Figure 3D) compared to simulated saccades (Figure 3E), is due to enhancement of responses for the short-delay condition.

In addition to comparing the mean spike rate in the long- and short-delay conditions, we also measured the neural response latencies. The difference in response latency between the long- and short-delay conditions following real saccades ranged from −10 to +10 ms (*SD* = 5 ms), tending on average to be shorter for the short-delay condition (mean reduction 0.9 ms; ASL = 0.02). The difference in response latency following simulated saccades ranged from −20 to +20 ms (*SD* = 10 ms). This variability was significantly greater than that following real saccades (Brown-Forsyth test, *P* = 0.003) and the mean difference was not significantly different from zero (mean increase 0.5 ms; ASL = 0.31).

### Enhancement of ocular following

While our primary objective was to investigate saccadic modulation of neural responses in MSTd, we also recorded all associated eye position signals. It is established that MSTd lies on the pathway that ultimately leads to short-latency ocular following (Kawano, 1999) and therefore analysis of the eye movement data plausibly offers an additional assay for assessing the relative contributions of internal as opposed to visual mechanisms underlying post-saccadic enhancement. We found that like the neural responses in MSTd, reflexive ocular following responses generated by the test stimulus were also enhanced after saccades. Figures 2C,D show example eye speed profiles from one monkey for both the short- and long-delay conditions for real and simulated saccades, respectively. Ocular following eye speeds were significantly greater in the short-delay condition than in the long-delay condition following both real and simulated saccades (Figures 2C,D, respectively; ASL < 0.001).

**Figure 4:**
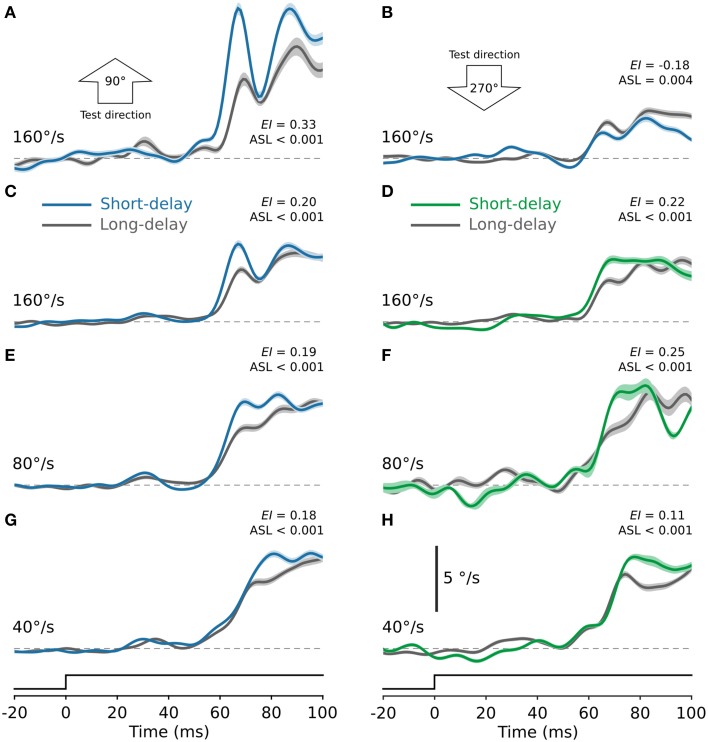
**Enhancement of ocular following eye speed after real and simulated saccades and the effect of test direction. (A,B)** Ocular following eye speed from one monkey for test stimuli presented after real saccades. In **(A)** the test stimuli were moving upward at 160°/s. Ocular following was robust for this test direction, and eye speed was significantly enhanced in the short-delay condition (blue) compared to the long-delay condition (gray). In **(B)** the test stimuli were moving downward at 160°/s. For this test direction ocular following was poor, with no evidence of post-saccadic enhancement. Ocular following was consistently poor for test stimuli moving in the downward direction. The remaining panels, **(C–H)**, show ocular following eye speed for test stimuli moving at 160, 80, and 40°/s, averaged across all directions tested. Panels on the left [**(C)**, 160°/s; **(E)**, 80°/s; **(G)**, 40°/s] show eye speed for test stimuli presented after real saccades. In all cases, ocular following eye speed was significantly enhanced in the short-delay condition (blue) compared to the long-delay condition (gray; ASL < 0.001). Panels on the right [**(D)**, 160°/s; **(F)**, 80°/s; **(H)**, 40°/s] show corresponding ocular following eye speed for test stimuli presented after simulated saccades. Again, in each case ocular following eye speed was significantly enhanced in the short-delay condition (green) compared to the long-delay condition (gray; ASL < 0.001). In all panels, solid lines show eye speed signals averaged across all trials of a given speed and the shaded regions indicate ±1 SE, estimated by bootstrapping.

In our experimental design the direction and speed of the test stimulus was determined by the tuning properties of the recorded neurons. It has been reported that ocular following is sensitive to both the speed and direction of the motion stimulus (Miles et al., 1986). Thus, while our stimuli were optimal for each recorded neuron, they were often sub-optimal for generating ocular following. Figure 4 shows ocular following responses from one animal in response to the test stimulus moving at a range of speeds. Figure 4A shows ocular following eye speed in response to a test stimulus moving upward (90°) at 160°/s, presented after real saccades. It is clear that initial ocular following eye speed is enhanced in the short-delay condition (blue) compared to the long-delay condition (gray; *EI* = 0.33, ASL < 0.001). However, we found that this enhancement was not evident for all test directions. For example, in the same animal ocular following was poor in response to the test stimulus moving in the opposite (270°, downward) direction, even for the same test speed of 160°/s. (Figure 4B). In fact, we consistently observed slower ocular following speeds and no post-saccadic enhancement for test stimuli moving in the downward direction. Nevertheless, Figures 4C–H show ocular following eye speed for three test speeds, 40, 80, and 160°/s, averaged across all directions tested. Panels on the left (Figures 4C,E,G) show ocular following eye speed for test stimuli presented after real saccades, while panels on the right (Figures 4D,F,H) show eye speed for test stimuli presented after simulated saccades. In all cases, initial ocular following eye speed is significantly enhanced in the short-delay condition compared to the long-delay condition (ASL < 0.001). Therefore, in contrast to the neural responses in MSTd, we found that enhancement of ocular following occurred after both real and simulated saccades.

## Discussion

The observation of post-saccadic enhancement of neural responses in MSTd, as quantified here, adds to a series of recent findings that have demonstrated modified neural activity in several primate brain areas at the time of saccades (Ibbotson and Krekelberg, 2011). First, there is a pre-saccadic suppression beginning around 30 ms before saccade onset. This suppression is maximal at saccade-onset and cannot be attributed to afferent visual input produced by the saccade (Ibbotson et al., 2008; Bremmer et al., 2009). Rather, this suppression is mediated by a central signal, a so-called *efference copy* or *corollary discharge* that arises during saccade planning (Wurtz, 2008; Bremmer et al., 2009). Second, immediately after the period of suppression there is a prolonged period of enhancement. Much like pre-saccadic suppression, a central mechanism associated with saccade generation could sensitize visual areas of the brain. For example, increased visual sensitivity in the post-saccadic period could arise from a corollary discharge associated with saccade generation, re-allocation of attention toward the new fixation target (Ibbotson and Krekelberg, 2011), or from residual eye position signals (Morris et al., 2012, 2013). Alternatively, displacement of the retinal image during saccades could activate visual gain control mechanisms that transiently enhance visual sensitivity. Previous experiments have demonstrated that post-saccadic enhancement of spontaneous neural activity occurs even in complete darkness (cat: Lee and Malpeli, 1998; monkey: Reppas et al., 2002; Ibbotson et al., 2008; Rajkai et al., 2008). While this is good evidence for an internal mechanism, modulation of spontaneous rate may not be linked directly to changes in sensitivity to visual stimulation. Therefore, before a mechanism can be identified a major question remains: does visual input during saccades have any influence on post-saccadic enhancement or does the phenomenon have an entirely internal origin?

Behavioral evidence for some visual enhancement has been reported by Kawano and Miles (1986). They showed that post-saccadic enhancement of ocular following eye speed was potentiated by strong visual stimulation during saccades, lending support to the notion of a visually mediated gain control mechanism underlying post-saccadic enhancement. Indeed, they went on to show that even a brief saccade-like image displacement could substantially enhance subsequent ocular following eye speed, even with no prior saccade. The enhancement of ocular following eye speed that we observed, following both real and simulated saccades, is therefore consistent with earlier behavioral reports.

In addition to the behavioral responses, we recorded spiking responses of visual neurons in area MSTd (an area known to play a role in reflexive ocular following). We delivered test stimuli after both real saccades and after simulated saccades. We found significant enhancement of neural responses following real saccades but not following simulated saccades. Furthermore, we found that there was significant enhancement following real saccades even in the absence of a patterned background during the saccades. Together these observations provide strong support for the notion that enhancement of visual sensitivity in MSTd following saccades is primarily mediated by an internal mechanism, not a visual mechanism.

As noted above, we also observed enhancement of neural responses after saccades over a blank background, but this condition produced less enhancement than that observed following saccades made over the high contrast background texture. This would seemingly suggest some additional enhancement of responses following saccades made over the high contrast background texture—a visual mechanism. However, we discount this interpretation of our data for the following reason: after saccades made over the blank screen, onset of the test stimulus was coincident with a step change in stimulus contrast (from 0% during the saccade to 100% at the onset of the test stimulus). Sudden appearance of a textured background such as we have used evokes robust firing from most cells in area MSTd (see for example Ibbotson et al., 2008). In our data, firing rates evoked by the test stimuli were greater on average (for both the long- and short-delay conditions) following saccades over the blank screen compared to those following saccades over the high contrast texture (rank sum tests, *P* < 0.004). We attribute this increase in firing to the sudden appearance of the stimulus—an “ON” response. As a result of this ON response, even in the absence of a saccade (i.e., in the long-delay condition) neurons are operating closer to their saturation point, leaving less capacity for any post-saccadic enhancement. Under these conditions it seems likely that our measure of enhancement is somewhat compromised, leading to the apparent reduction in the level of enhancement observed. From these combined results, we can conclude two things. First, saccade-like displacement of the background texture in the absence of a real saccade does not generate enhancement of neural responses. Second, real saccades across both textured and blank backgrounds influence the visual system such that responses to subsequent image motion are significantly enhanced.

The discrepancy which we observe between the neural and behavioral metrics suggests that although MSTd has been implicated in mediating ocular following (Kawano, 1999) it is unlikely to be the only pathway involved. Indeed, it is known that other brain areas such as the nucleus of the optic tract (NOT), a sub-cortical area associated with the accessory optic system, contribute to short-latency ocular following (Inoue et al., 2000) and contain neurons tuned to detect saccade-like image displacements (Ibbotson and Mark, 1994). Therefore, the visual enhancement of eye speeds described by Kawano and Miles (1986), and confirmed here, is likely mediated by some parallel neural pathway external to MSTd.

## Author contributions

NC, MM, and MI conceived and designed the experiments; NC, MM, and MI performed the experiments; SC contributed unpublished analytic tools; SC analyzed the data; all authors interpreted the results of the experiments; SC and MI drafted the manuscript; SC and MI prepared the figures; All authors edited and revised the manuscript and approved the final version.

## Funding

This work was supported by the Australian Research Council through the Centre of Excellence in Vision Science (CE0561903) and the Centre of Excellence for Integrative Brain Function (CE140100007), the National Health and Medical Research Council of Australia (224263, 525461), the National Institutes of Health (EY06069/EY/NEI NIH HHS/United States, P51 OD010425/OD/NIH HHS/United States), and by Research to Prevent Blindness.

### Conflict of interest statement

The authors declare that the research was conducted in the absence of any commercial or financial relationships that could be construed as a potential conflict of interest.
